# Selective Ensemble Based on Extreme Learning Machine for Sensor-Based Human Activity Recognition

**DOI:** 10.3390/s19163468

**Published:** 2019-08-08

**Authors:** Yiming Tian, Jie Zhang, Lingling Chen, Yanli Geng, Xitai Wang

**Affiliations:** 1 School of Artificial Intelligence, Hebei University of Technology, Tianjin 300130, China; 2 School of Engineering, Merz Court, Newcastle University, Newcastle upon Tyne NE1 7RU, UK; 3 National Research Center for Rehabilitation Technical Aids, Beijing Key Laboratory of Rehabilitation Technical Aids for Old-Age Disability, Key Laboratory of Human Motion Analysis and Rehabilitation Technology of the Ministry of Civil Affairs, Beijing 100176, China

**Keywords:** human activity recognition, selective ensemble, wearable sensor, extreme learning machine, diversity measure, glowworm swarm optimization

## Abstract

Sensor-based human activity recognition (HAR) has attracted interest both in academic and applied fields, and can be utilized in health-related areas, fitness, sports training, etc. With a view to improving the performance of sensor-based HAR and optimizing the generalizability and diversity of the base classifier of the ensemble system, a novel HAR approach (pairwise diversity measure and glowworm swarm optimization-based selective ensemble learning, DMGSOSEN) that utilizes ensemble learning with differentiated extreme learning machines (ELMs) is proposed in this paper. Firstly, the bootstrap sampling method is utilized to independently train multiple base ELMs which make up the initial base classifier pool. Secondly, the initial pool is pre-pruned by calculating the pairwise diversity measure of each base ELM, which can eliminate similar base ELMs and enhance the performance of HAR system by balancing diversity and accuracy. Then, glowworm swarm optimization (GSO) is utilized to search for the optimal sub-ensemble from the base ELMs after pre-pruning. Finally, majority voting is utilized to combine the results of the selected base ELMs. For the evaluation of our proposed method, we collected a dataset from different locations on the body, including chest, waist, left wrist, left ankle and right arm. The experimental results show that, compared with traditional ensemble algorithms such as Bagging, Adaboost, and other state-of-the-art pruning algorithms, the proposed approach is able to achieve better performance (96.7% accuracy and F1 from wrist) with fewer base classifiers.

## 1. Introduction

In recent years, many works [1,2] have shown that human activity recognition (HAR) has enabled various applications. For instance, daily activities may provide information for health conditions of human beings, and some diseases, such as cerebral small vessel disease [3] and stroke [4], have been proved to be related to the mobility of the human body. Therefore, HAR has been utilized to detect some diseases. In addition, the HAR system can obtain the users’ daily energy expenditure, which can be utilized as a reference for their exercise advice. Moreover, sports training such as swimming [5] and badminton [6] also benefits from HAR. According to the types of data acquisition devices employed, HAR can be divided into vision-based and sensor-based approaches. Vision-based approaches recognize different activities by using video or image sequences. Although vision-based approaches have experienced great breakthroughs in recent years, they still suffer from some drawbacks, including privacy, pervasiveness and complexity [7]. With the development of microelectronics, sensor-based approaches that make use of sensor readings from accelerometers, gyroscopes and magnetomers have attracted more attention around the world. These three kinds of sensor have been utilized in a lot of studies [1,2,5,6], demonstrating their advantages and superior performance in HAR.

A lot of machine learning algorithms have been explored for sensor-based activity recognition. In [8], a neural network was utilized for recognizing three states of activity, including static, transition and dynamic state and 15 kinds of activities. The neural network was applied to recognize eight different activities of construction workers, and showed the best recognition accuracy when compared with five other machine learning algorithms [9]. KNN was also utilized to recognize everyday activities in [10], and a 99.01% overall accuracy was reported in their experiments. In [11], a decision tree (DT) classifier was applied to the detection of activity intensity in youth with cerebral palsy. The computationally efficient support vector machine (SVM) classifier has also been applied in HAR. Wu et al. [12] utilized KNN and SVM as classifiers to demonstrate the proposed features and feature selection method in HAR. By using coordinate transformation and principal component analysis, an online-independent support vector machine (OISVM) [13] has showed that it is effective in improving the robustness of HAR system. Since experimental conditions such as the datasets and extracted features are different, it is difficult to compare the performances of the above classifiers.

The recently proposed extreme learning machine (ELM) [14] is an effective efficient learning algorithm based on single-layer feedforward network (SLFN). It has many advantages, including a simple structure, faster learning rate, and better generalization ability. Therefore, ELM has been widely used in HAR in recent years. In [15], ELM was applied to realize location-adaptive activity recognition; due to the advantages of ELM, experiments showed that the proposed model could adapt the classifier to new device locations quickly. Xiao et al. [16] proposed kernel Fisher discriminant analysis (KDA)-based ELM classifier to recognize six kinds of activity, the experiments showed that it could achieve higher accuracy and faster learning speed than the BP and SVM. An ELM ensemble learning algorithm called average combining extreme learning machine (ACELM) was proposed by [17] to construct a more stable classifier. Moreover, several different variants of ELM have also been proposed and applied in problems of HAR, such as the imbalanced datasets problem [18,19], class incremental learning [20], and cross-person activity recognition [21,22]. However, due to its simple structure and the randomly generated hidden layer parameters, including input weights and hidden layer bias values, a single ELM classifier usually produces unstable outputs, especially when the testing data and the training data are very different in distribution [23].

Ensemble learning has primarily been considered for improving the generalization performance and recognition accuracy of a single classifier. The ensemble learning algorithm was first proposed by Hansen et al. [24]. Their research shows that the ensemble of multiple neural networks can improve the generalization performance of neural networks. Currently, Bagging and Boosting are the two most popular ensemble algorithms. Despite the significant progress of ensemble learning, the accuracy improvement is not proportional to the number of base classifiers. Furthermore, an ensemble learning algorithm that produces too many base classifiers may lead to large computational complexity and low efficiency. Selective ensemble, which is also known as ensemble pruning, is an approach for addressing these issues. In general, the set of base classifiers determined by ensemble pruning tries to meet the performance criterion of maximizing the recognition accuracy and minimizing computation time. If a classifier pool contains *M* base classifiers, 2*^M^*-1 nonempty base classifier subsets can be generated. This makes selecting a subset of classifier with the optimal performance to be an NP-complete problem [25].

To improve the performance of the system, many ensemble pruning approaches have been proposed, and these methods can be categorized into three main groups: ordering-based, optimization-based, and clustering-based pruning approaches [26]. Ordering-based pruning is the most widely used algorithm. For example, two selective techniques for multiple neural networks: forward selection and backward elimination were proposed by Ahmad and Zhang [27,28] to improve model generalization. Li et al. [29] proposed a maximum relevance and minimum redundancy-based ensemble pruning (MRMREP) method for ensemble learning-based facial expression recognition. The proposed method utilized two important factors (the correlation between target labels and predictions, the redundancy between classifiers) to order all base classifiers. Through the experiment, the proposed MRMREP can achieve superior results compared with other ensemble pruning methods. Cao et al. [30] designed a multi-sensor fusion with ensemble pruning system (MSF-EP) for activity recognition and presented four ordering-based ensemble pruning methods to optimize the multi-sensor deployment. A novel ordering-based metric named the margin and diversity-based measure (MDM) was proposed by [31] to explicitly evaluate the importance of base classifiers. Comparative experiments with the other state-of-the-art ensemble pruning methods proved the effectiveness of the algorithm.

Optimization-based pruning has also attracted tremendous attention from scholars. Zhou [32] proposed a genetic algorithm-based selective ENsemble (GASEN) approach that utilized the genetic algorithm (GA) to evolve the weights of base neural networks. According to the evolved weights of base neural networks, it selects some neural networks with higher prediction accuracy and a large diversity between each other and to make up the ensemble. The experiments showed that it has stronger generalization ability compared with some popular ensemble approaches such as Bagging and Boosting. Zhu et al. [33] proposed an optimization-based pruning method based on improved discrete artificial fish swarm algorithm (IDAFSA), which utilized an artificial fish swarm algorithm as an optimization strategy to find the optimal classifier subset instead of the GA. Experimental studies on 29 datasets from the UCI provide the effectiveness of the algorithm. In [34], a bee algorithm (BA) was utilized to select the optimal ensemble subset from a pool of different base classifiers including support vector machine, k-nearest neighbor and linear discriminant analysis classifiers. The proposed method can achieve 83% of accuracy, 93% of specificity and 60% of sensitivity in the mammogram.

The clustering-based pruning approaches are derived from clustering techniques. This method mainly includes two steps: Firstly, the base classifiers in the ensemble are divided into different clusters. The classifiers from the same cluster have similar classification results, while the classifiers from different clusters perform in a more diverse manner. Nowadays, several clustering techniques are utilized in ensemble pruning, including k-means [35], hierarchical agglomerative clustering [36], and deterministic annealing [37]. Finally, in order to increase the diversity of the ensemble, we obtain the base classifier in different clusters. For example, Bakker et al. [38] utilized the classifiers at the centroid of each cluster to constitute the final ensemble.

Although there are many HAR studies based on ensemble learning technology [39,40,41,42,43,44], to our best knowledge, there is still no work attempting to improve the performance of HAR through a selective ensemble approach. Most of the ensemble learning-based HAR studies [17,30,39] combined all the trained base classifiers for recognition. However, some base classifiers may be redundant and have poor performance, which may affect the performance of the recognition system. Therefore, a selective ensemble-based approach may be a good choice for improving the performance of ensemble-based HAR. As a traditional kind of ordering-based pruning method, pairwise diversity can be utilized to measure the diversity among base classifiers and shows good performance in many research works when utilized as a strategy for pre-pruning base classifiers [45,46]. Additionally, glowworm swarm optimization (GSO) is a biomimetic optimization algorithm [47] that has advantages of fast convergence speed and good global convergence. It has been utilized in multiple-objective environmental economic dispatch [48], sensor deployment [49], and vehicle routing problems [50]. Compared with GSO, other heuristic algorithms, such as the genetic algorithm, can also successfully solve the ensemble pruning problem. However, when the number of base classifiers increases, other heuristic algorithms will encounters problems when solving the ensemble pruning problem, including poor solution quality, large time consumption, and low convergence. Based on these considerations, this paper proposes a novel selective ensemble method, DMGSOSEN, which combines pairwise diversity and the GSO algorithm for HAR. Firstly, considering the diversity of base classifiers in the initial pool of ensemble, bootstrap sampling is utilized to train base ELMs. Secondly, we utilize pairwise diversity measures for each base classifier to pre-prune the base ELMs. This step can preserve the base classifier with large diversity, eliminate the redundant base classifier and reduce the complexity of the GSO-based pruning stage. Finally, further pruning is carried out by using the GSO method and the remaining base ELMs are integrated by majority voting.

The contributions of this paper can be described as follows:

(1) We propose a novel sensor-based HAR approach based on ELM and DMGSOSEN for improving the recognition performance and reducing the size of ensemble. The DMGSOSEN is a novel ensemble pruning approach that combines existing algorithms, it has good capacity of selecting the generated base classifiers to show its desirable performance for HAR.

(2) We find that the double-fault measure has better performance when compared with four other pairwise diversity measures. Based on the double-fault measure pre-pruning, we utilize discrete glowworm swarm optimization algorithm to further search the optimal sub-ensemble.

(3) The DMGSOSEN-based approach could select superior base classifiers adaptively through optimization algorithm, which makes it more practicable to deal with the various styles of activity.

(4) We demonstrate the efficiency of the proposed DMGSOSEN-based HAR approach with dataset acquired from different body positions.

The remainder of this paper is organized as follows: In Section 2, we present details of the proposed HAR approach based on ELM and DMGSOSEN. The DMGSOSEN is a novel combination of existing algorithms for ensemble pruning in ensemble learning-based HAR. Section 3 and Section 4 describe the experimental dataset and experimental setup, respectively. Following that, comparative experiments are carried out to validate the effectiveness of the proposed approach in Section 5. Finally, we draw conclusions in Section 6.

## 2. The Proposed HAR Approach Based on ELM and DMGSOSEN

**Figure 1:**
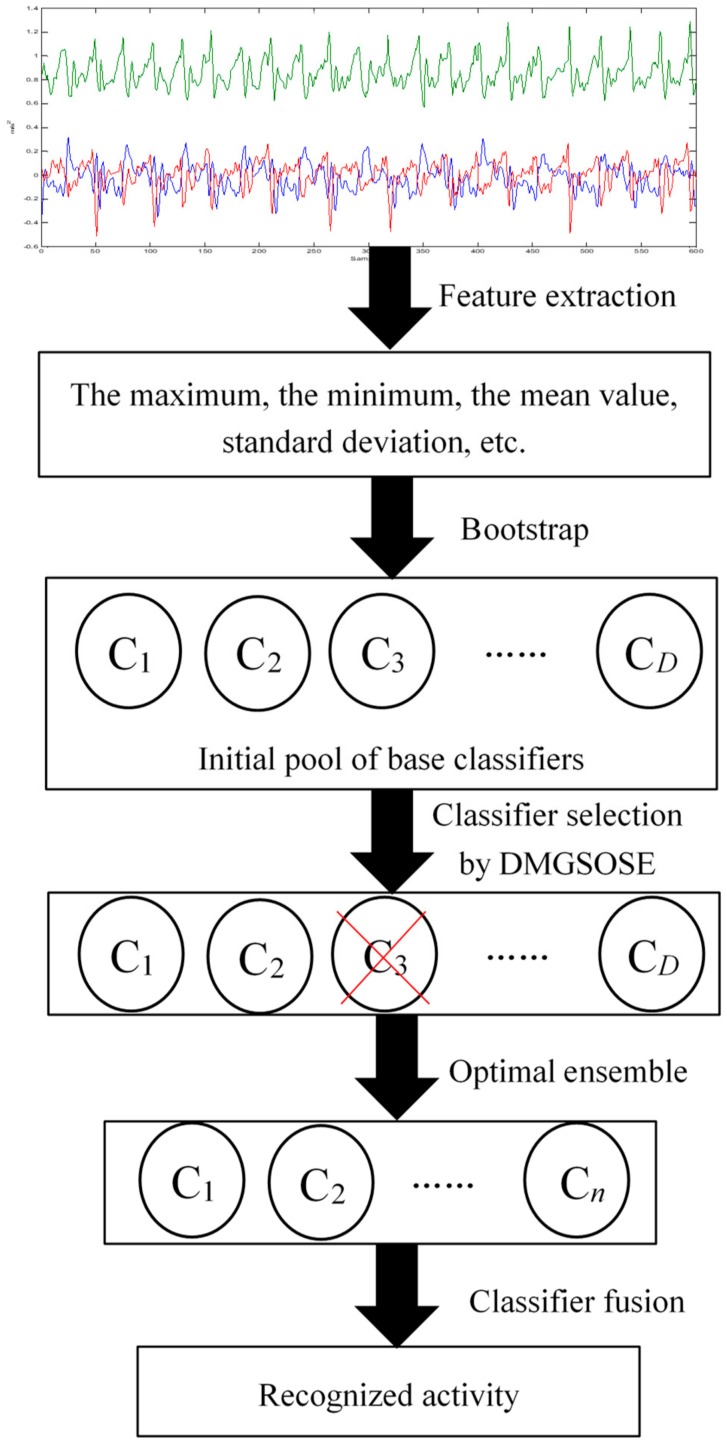
The framework of proposed selective ensemble-based HAR approach.

Figure 1 shows the architecture of the proposed HAR approach. As shown in Figure 1, the proposed selective ensemble learning method for HAR contains three modules: base classifier generation, base classifier selection by DMGSOSEN, and classifier fusion. First, the initial pool of base classifier is constructed through bootstrap sampling. Because there may be poor performance and redundant base classifiers in the initial pool, we will not utilize all the base classifiers to establish an ensemble recognition system. Second, the DMGSOSEN is proposed to select superior individual classifiers from the initial pool of base classifier and optimize the ensemble. The DMGSOSEN combines double-fault measure and GSO algorithm uniquely for base classifier selection in HAR. Third, majority voting is utilized to integrate the selected base classifiers. In the following subsections, we describe details of these three modules.

### 2.1. Base Classifier Generation

**Figure 2:**
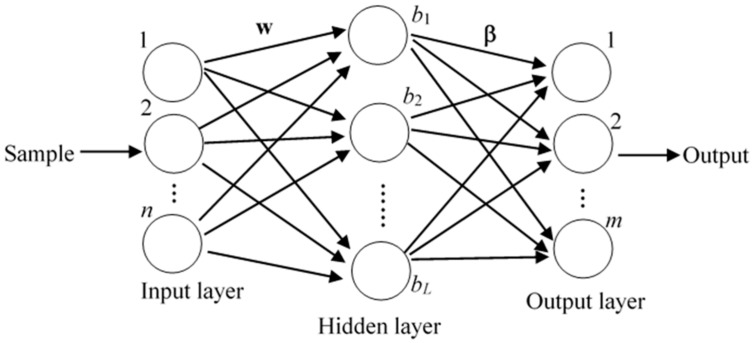
The basic structure of ELM.

In this paper, ELM is selected as base classifier of the ensemble system due to its simple structure and good generalization performance. The basic structure of ELM has input, hidden and output layer nodes, which is shown in Figure 2. The only parameter that needs to be set is the number of hidden layer nodes. For any *N* different samples (**x***_j_*, **t***_j_*), *j* = 1, 2, …, *N*, where $x_{j}={\left[x_{j1},x_{j2},\cdots ,x_{\mathrm{jn}}\right]}^{T}$ is the *j*th sample, each sample contains *n*-dimensional features, and $t_{j}={\left[t_{j1},t_{j2},\cdots ,t_{\mathrm{jm}}\right]}^{T}$ is the encoded class label. All samples belong to *m* different classes and the ELM mathematical model with *L* hidden neurons can be expressed as:

(1) $$\sum_{i=1}^{L}\beta_{i}g(w_{i}\cdot x_{j}+b_{i})=t_{j},\;j=1,2,\cdots ,N$$

where *g*(*x*) is the excitation function, **w***_i_*, *b_i_*, and **β***_i_* are the input weight, hidden layer bias and output weights of the *i*_th_ hidden neuron node respectively. Equation (1) can be written in matrix form:

(2) Hβ=T

where **β** represents the output weight, **T** is the corresponding coding class label, and **H** is the hidden layer output matrix:

(3) $$H={\left[\begin{array}{ccc} g(w_{1}\cdot x_{1}+b_{1}) & \cdots & g(w_{L}\cdot x_{1}+b_{L})\\ \vdots & \cdots & \vdots \\ g(w_{1}\cdot x_{N}+b_{1}) & \cdots & g(w_{L}\cdot x_{N}+b_{L}) \end{array}\right]}_{N\times L}$$

The output weight **β** can be calculated by Equation (4):

(4) $$\beta =H^{†}T$$

where **H**^†^ is the generalized inverse matrix of H.

HAR requires the recognition system to not only have good recognition accuracy, but also to have good generalization ability. Although ELM can handle general recognition problems, different subject-related features and various styles of a certain activity usually degrade the performance of the ELM. Furthermore, the generalization ability of an algorithm is usually influenced by training samples with category representations that can determine the decision boundaries of different activity categories. The ensemble learning techniques can be utilized to improve the generalization ability of a single classifier. Diversity is an important principle for base classifier generation, it is of great significance for improving the generalization ability of ensemble learning. Bootstrap and bagging have been utilized in several studies [27,28,33] to improve the diversity of base classifiers for enhancing the generalization ability of ensemble. In this paper, the bootstrap sampling method is utilized to obtain the training dataset for each ELM.

### 2.2. Pairwise Diversity Measures

The diversity between the base classifiers is a key factor in determining the performance of an ensemble system. The diversity measure between the base classifiers is not simple, despite a lot of theories have been proposed to measure the diversity among base classifier, there is currently no uniform definition of diversity among classifiers. Considering the small computational complexity of pair-based diversity measures and their good performances on ensemble pruning, five pairwise diversity measures which belongs to ordering-based method will be compared with respect to their pre-pruning performances. We will choose the pairwise diversity measure with the best performance for DMGSOSEN. These five methods will be described as follows:

Disagreement [51] was proposed by Skalak based on the concept of diversity. The larger the disagreement measure, the greater the diversity between the base classifiers. The disagreement measure for the two base classifiers *C_i_* and *C_j_* can be calculated by the following formula:

(5) $${\mathrm{Dis}}_{\mathrm{ij}}=\frac{b+c}{a+b+c+d}$$

where *d* represents the number of samples when classifier *C_i_* and *C_j_* recognize errors, *a* represents the number of samples when classifier *C_i_* and *C_j_* recognize correctly, *b* represents the number of samples when classifier *C_i_* recognizes errors while classifier *C_j_* recognizes correctly, *c* represents the number of samples when classifier *C_i_* recognizes correctly while classifier *C_j_* recognizes errors.

Correlation coefficient [52] is derived from statistics, and the correlation coefficient of two classifiers can be calculated by Equation (6).

(6) $$\rho_{\mathrm{ij}}=\frac{ad-bc}{\sqrt{\left(a+b)(c+d)(a+c)(b+d\right)}}$$

The Q-statistic was proposed by Yule [53], and can be regarded as a simplified operation of the correlation coefficient. It is defined by Equation (7).

(7) $$Q_{\mathrm{ij}}=\frac{ad-bc}{ad+bc}$$

The Kappa-statistic is widely used in statistics and it was used to analyze the diversity between classifiers for the first time by Margineantu and Dietterich [54]. The amount of computation using paired Kappa is less than the Q-statistic measure. The smaller the paired Kappa measure, the smaller the correlation of the base classifier. The formula is as shown in Equation (8).

(8) $$K_{\mathrm{ij}}=\frac{2(ad-bc)}{\left(a+b)(b+d)+(a+c)(c+d\right)}$$

Giacinto and Roli proposed a double-fault measure in 2001 [55], which can be utilized to calculate the proportion of samples misclassified by both classifiers. It can be expressed as Equation (9).

(9) $${\mathrm{DF}}_{\mathrm{ij}}=\frac{d}{a+b+c+d}$$

### 2.3. Discrete Glowworm Swarm Optimization

After pre-pruning the base classifier based on the pairwise diversity measures, this paper utilizes the GSO to select base classifiers with better performance to optimize the sub-ensemble. As the traditional GSO is proposed for continuous optimization problems, it is not suitable for selective ensemble which belongs to a discrete combinatorial optimization problem. To make GSO suitable for dealing with discrete problems in binary space, the discrete glowworm swarm optimization (DGSO) is detailed in this section. First, the GSO algorithm is briefly described.

#### 2.3.1. GSO

GSO is a heuristic algorithm inspired by mimicking the luminescent behavior of glowworms in nature. In GSO, glowworms are randomly distributed throughout the entire search space with a certain amount of fluorescein. In the range of the field of view, glowworms constantly move closer to those that are brighter than themselves, thus achieving group optimization and finally converging on the global optimal solution. The basic steps are as follows:

Step 1: Initialization of algorithm parameters.
Step 2: Convert the fitness value *J*(***x****_i_* (*t*)) corresponding to the position ***x****_i_*(*t*) of glowworm *i* at time *t* to the fluorescein value *l_i_*(*t*) by Equation (10).
  (10) $$l_{i}(t)=(1-\rho )l_{i}(t-1)+\gamma J(xi(t))$$
  where *ρ* is the fluorescein decay constant belonging to (0, 1) and the *γ* is the fluorescein enhancement constant.
Step 3: Each glowworm selects a neighborhood set *N_i_*(*t*) whose individual brightness is higher than itself in its dynamic decision domain radius $r_{d}^{i}(t)$ $\left(0<r_{d}^{i}(t)<r_{s}\right)$.
Step 4: Calculate the probability *p_ij_*(t) of the movement of individual *i* to the individual *j* (*j* ∈ *N_i_* (*t*)) in its neighborhood set *N_i_*(*t*) by Equation (11).
  (11) $$p_{\mathrm{ij}}(t)=\frac{l_{j}(t)-l_{i}(t)}{\sum_{k\in Ni(t)}l_{k}(t)-l_{i}(t)}$$
Step 5: Select the moving object and update the glowworm position according to Equation (12).
  (12) $$x_{i}(t+1)=x_{i}(t)+s(\frac{xj(t)-x_{i}(t)}{\left\|x_{j}(t)-x_{i}(t)\right\|})$$
  where *s* > 0 is the step that one glowworm is moving towards the other.
Step 6: Update the dynamic decision radius of glowworm by Equation (13).
  (13) $$r_{d}^{i}(t+1)=\min(r_{s},\max(0,r_{d}^{t}(t)+\beta (nt-\left|N_{i}(t)\right|)))$$
  where *β* is a constant and *n_t_* is a parameter used to control the number of neighbors.

#### 2.3.2. DGSO

Based on the traditional GSO algorithm, the modifications of DGSO mainly include the following aspects: the encoding method of the solution, the position update method of the glowworm and the construction of the fitness function. Through these improvements, DGSO is able to search in a binary discrete space. These modifications will be detailed in this section.

(a) Encoding method

When using DGSO to solve the selective selection problems, the structure of the solution can be expressed by:

(14) $$x_{i}(t)=[x_{i1}(t),x_{i2}(t),\cdots ,x_{\mathrm{ih}}h(t),\cdots ,x_{\mathrm{iD}\prime }(t)]$$

The above formula indicates the position of the *i*th glowworm in the *t*th iteration and *D*′ represents the dimension of each glowworm in the population, that is, the number of base classifiers after pre-pruning by diversity measures. In selective ensemble, *x_ih_* can only be 0 or 1, *x_ih_*(*t*) = 1 indicates that the *i*th glowworm selects the *h*th base classifier in the *t*th iteration and *x_ih_*(*t*) = 0 means that the *i*th glowworm does not select the *h*th base classifier in the *t*th iteration.

The initial position of the glowworm is obtained by:

(15) $$x_{\mathrm{ih}}(0)=\left\{\begin{array}{ll} 1 & rand\geq 0.5\\ 0 & rand<0.5 \end{array}\right.$$

where *rand* is a randomly number generated from (0, 1).

(b) Glowworm position update

The fixed step search method is not suitable for DGSO in binary discrete space. To make the search process of the discrete GSO algorithm simple and efficient, this paper selects the position update formula according to probability. In the *t*th iteration of the DGSO algorithm, current glowworm position can be expressed as $x_{i}(t)=[x_{i1}(t),x_{i2}(t),\cdots ,x_{\mathrm{iD}\prime }(t)]$ and target glowworm position can be expressed as $x_{j}(t)=[x_{j1}(t),x_{j2}(t),\cdots ,x_{\mathrm{jD}\prime }(t)]$. When the position update is performed, each dimension variable in the individual position row vector is updated with a certain probability, thereby realizing the update of the entire vector which is the position update. The specific position update formula is as follows:

(16) $$x_{\mathrm{ik}}(t+1)=\left\{\begin{array}{l} x_{\mathrm{ik}}(t),\;r(k)\leq p_{1}\\ x_{\mathrm{jk}}(t),\;p_{1}<r(k)\leq p_{2}\;\\ \left|x_{\mathrm{ik}}(t)-1\right|,\;r(k)>p_{2} \end{array}\right.$$

where ***r*** is a randomly generated *D*′-dimensional vector $r=\{r_{1},r_{2},\cdots ,r_{k},\cdots ,r_{D\prime }\}$ and *r_k_* ∈ [0,1], *p*_1_ and *p*_2_ ∈ [0,1] are both selected parameters for the update formula.

(c) Infeasible solution

When the elements in the solution vector appear to all be 0 or all be 1, these two cases correspond to the selective ensemble system containing no base classifiers and all base classifiers, respectively. Therefore, both of the above cases are considered to be infeasible solutions. For both cases, Equation (15) is utilized in this paper to randomly generate feasible solutions to improve search efficiency.

(d) Distance between glowworms

Since selective ensemble learning is a discrete combination optimization problem and the solution vector of discrete GSO only contains two values of 0 and 1, the traditional Euclidean distance is not suitable for calculating the distance between glowworm. Therefore, the Hamming distance metric is utilized in this paper instead, it is the number of different characters in the same position corresponding to two equal length vectors. If the positions of the individual glowworm *i* and *j* in the *t*th iteration are:

(17) $$x_{i}(t)=(x_{i1},x_{i2},\cdots ,x_{\mathrm{iD}\prime }),\;x_{j}(t)=(x_{j1},x_{j1},\cdots ,x_{\mathrm{iD}\prime })$$

Then the distance between the individual glowworm *i* and *j* at the *t*th iteration is recorded as:

(18) $$hm\_d_{\mathrm{ij}}(t)=hamming\_distance(x_{i}(t),x_{j}(t))$$

(e) Fitness function

The fitness function of the selective ensemble problem can be defined as:

(19) $$F_{n}=\frac{1}{m}\sum_{j=1}^{m}Acc(f(x_{j}),y_{j})$$

where *F_n_* is the recognition accuracy between the recognized category and the actual category, $Acc(f(x_{j}),y_{j})=\left\{\begin{array}{ll} 1, & \;\mathrm{if}\;f(x_{j})=y_{j}\\ 0, & \;\mathrm{if}\;f(x_{j})\neq y_{j} \end{array}\right.$, *m* represents the number of test samples, *f*(*x_j_*) and *y_j_* represent the recognized category and actual category on the *j*th test sample, respectively. The higher the fitness value, the higher the selective ensemble accuracy.

### 2.4. The Proposed DMGSOSEN-Based Classifier Selection

Step 1: Establish an initial pool of base ELMs. In this paper, bootstrap sampling is utilized to generate *D* training subsets *S_i_*, *i* = 1, 2, ..., *D*. The base ELM is trained on each subset *S_i_*, so a base classifier pool with *D* ELMs can be obtained.
Step 2: Pre-prune the base classifier pool based on pairwise diversity measures. For ensemble selection, it is not only computationally expensive but also difficult to search for the optimal sub-ensemble when using an optimization-based pruning method, especially when the initial pool of base classifiers is large in size. To tackle this problem, we pre-prune the initial base ELMs pool in order to reduce the number of base classifiers before using the GSO method. The base classifiers that make up the ensemble system should not only have good performance, but also have great diversity, in order to ensure good generalization ability of ensemble system. Thus, the five kinds of pairwise diversity measures mentioned in Section 2.2 are respectively utilized to calculate the diversity of each base ELM and eliminate the base ELMs with small diversity in the base classifier pool. The performances of the five pairwise diversity measures will be compared, and we will choose the best one as the evaluative criteria. The pairwise diversity measure of each base ELM can be obtained by:
  (20) $${\mathrm{Div}}_{i}=\frac{1}{D}\sum_{j=1}^{D}{\mathrm{div}}_{\mathrm{ij}}$$
  where *Div_i_* means the pairwise diversity measure of the *i*th base ELM, *div_ij_* represents the pairwise diversity measure between the *i*th base ELM and the *j*th base ELM, 1 < *i* ≠ *j* ≤ *D*.
Step 3: DGSO pruning. After pre-pruning by pairwise diversity measure, the *D* base classifiers in the base classifier pool retain the *D*′ base classifiers. Next, the GSO algorithm is used to continue pruning the *D*′ base classifiers.
  Step 3.1: Initialize the basic parameters of the DGSO. These parameters include population size *g*, maximum iteration number *iter*_*max*, fluorescein volatilization factor *ρ*, fluorescein update rate *γ*, dynamic decision domain update rate *β*, threshold *n_t_* of glowworm contained in the neighborhood set *N_i_*(*t*), initial fluorescein value *l*_0_, initial dynamic decision radius $r_{d}^{i}(0)$, perceived radius *r_s_*, initial solution ***x***(0), the parameters of position update formula: *p*1, *p*2.
  Step 3.2: A set of glowworms in initial positions can been obtained. Calculate the fitness value of the glowworm according to Equation (19) and the corresponding fluorescein value by Equation (10). The fitness value *J*(*****x******_i_*(*t*)) corresponding to the position *****x******_i_*(*t*) of the glowworm *i* at the *t*th iteration is converted to fluorescein value *l_i_*(*t*). Save the glowworm’s position with the maximum fitness function *F_max_*.
  Step 3.3: Calculate the Hamming distance between glowworm individuals by Equation (18). Each glowworm selects a neighbor set *N_i_*(*t*) whose fluorescein values are larger than itself in its dynamic decision domain radius $r_{d}^{i}(t)(0<r_{d}^{i}(t)\leq r_{s})$.
  Step 3.4: Calculate the probability *p_ij_*(*t*) of the glowworm *i* moving to the individual *j*(*j* ∈ *N_i_*(*t*)) in the neighborhood set *N_i_*(*t*) by Equation (11) and select the moving object by the roulette method according to the probability.
  Step 3.5: Randomly generate a *D*′-dimensional vector ***r*** between 0 and 1, and $r(k)\in \{r_{1},r_{2},\cdots ,r_{k},\cdots ,r_{D\prime }\}$. According to the value of *r*(*k*), update the position of each candidate glowworm by Equation (16).
  Step 3.6: In the set of glowworms in new positions, the glowworm’s position with the maximum fitness function is *F*’*_max_*. If *F*’*_max_* > *F_max_*, set *F_max_* = *F*’_max_, Otherwise, *F_max_* = *F*_max._ Update the dynamic decision domain radius of the glowworm individual according to Equation (13).
  Step 3.7: Check termination criteria. If the maximum number of iterations is not reached, return to Step 3.2. Otherwise, go to Step 4.
Step 4: Lastly, the glowworm with the best fitness value is considered for majority voting. Then, the base ELMs participating in the final ensemble can be acquired, which corresponds to the coding combination with the fitness value. The final recognition result is obtained by majority voting: *f*(x) = arg max *N_i_*, *N_i_* is the number of base classifiers that the sample x is recognized as the *i*th activity.

**Figure 3:**
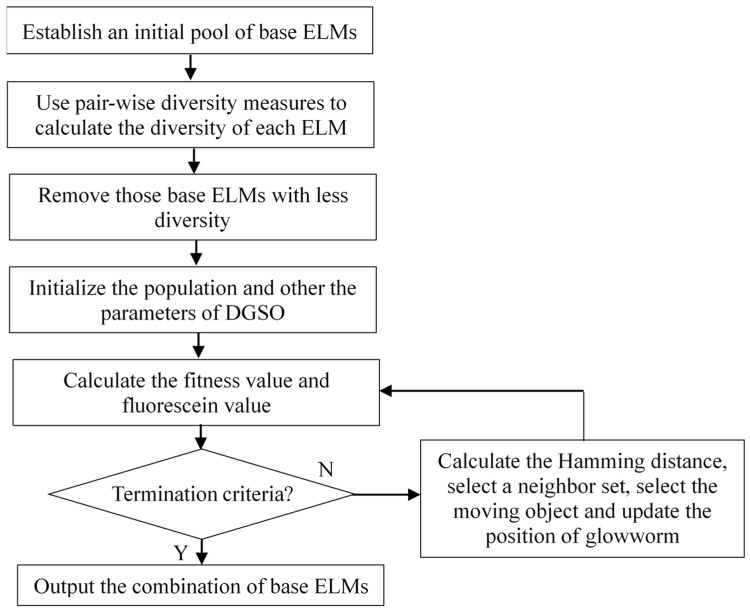
Workflow of the DMGSOSEN.

The flowchart of the proposed DMGSOSEN approach is illustrated in Figure 3.

## 3. Experimental Dataset and Feature Extraction

### 3.1. Dataset

**Figure 4:**
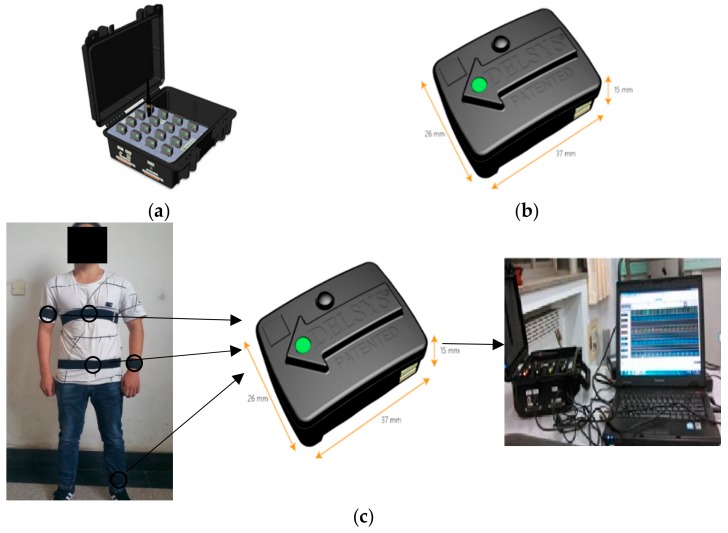
Human activity data acquisition platform based on acceleration sensor: (**a**) the data acquisition platform, (**b**) data collection node containing a triaxial accelerometer, (**c**) experimental data acquisition process.

**Figure 5:**
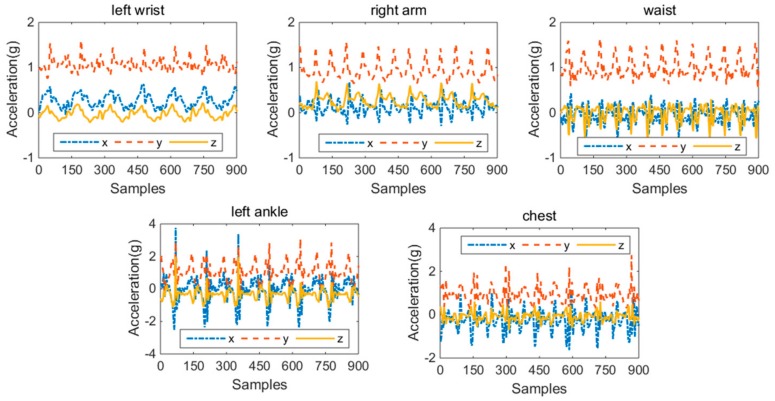
The triaxial accelerometer data of “walking” from the chest, waist, left wrist, left ankle and right arm.

In the experiment, we acquired the dataset in our laboratory by using the TRIGNO^TM^ wireless system from Delsys Company, as shown in Figure 4. The TRIGNO^TM^ wireless system contains a data acquisition platform and a collection node, which are shown in Figure 4a,b, respectively. The collection node integrates a triaxial accelerometer which has a sampling frequency of 150 Hz and an acceleration range of ±6 G with a resolution of 0.016 (G is the gravitational constant). Figure 4c presents the fixed position of the collection node and the workflow of the system implementation. Since the experimental platform has wireless transmission function, the acceleration signal can be transmitted to the data acquisition platform from collection node. The ZigBee protocol is utilized in the study. Once received by the acquisition platform, the data are transmitted and stored in the computer. Five healthy students, including 3 males and 2 females, participated in the data collection. Their ages ranged from 20 to 34, and their average age was 26. Each participant was asked to fix the collection node to five different body parts: chest, waist, left wrist, left ankle and right arm. Before the start of each experiment, we utilized straps to fix the sensors on the body and checked the sensors were in the same position as the previous subject. The activities performed by each subject included walking, running, going upstairs, going downstairs, jumping and standing. These activities were separated and there were no transitions. Therefore, a dataset with five sensor locations could be obtained. Figure 5 shows the activity data of “walking” from the selected five positions. The preprocessing of the acceleration signal includes removing abnormal data and signal denoising. Data points with numerical anomalies in the acceleration signal sequence were removed. Discrete wavelet transform was adopted to filter out noise signals in this paper and the wavelet function Coif5 was utilized to filter out noise signals from acceleration signals. Then, the sliding window was utilized to divide the acceleration signal after preprocessing; 300 samples were chosen as the window length, and a 50% overlap between adjacent windows was adopted.

### 3.2. Feature Extraction

After using sliding window to divide the triaxial acceleration data, we extracted features from these windows. These features include the maximum, the minimum, the mean value, standard deviation *σ*, skewness *S*, kurtosis *K*, correlation coefficient *C* between three axes, signal magnitude area (SMA), and number of zero crossings which is number of zero crossings in a window after subtracting the window mean value from every window sample. Various research works have proven the effectiveness of these features on HAR [8,13,16,30]. These features can be expressed as follows:

(21) $$mean=\frac{1}{N}\sum_{i=1}^{N}a_{i}$$

(22) $$\sigma =\sqrt{\frac{1}{N}\sum_{i=1}^{N}{\left(a_{i}-mean\right)}^{2}}$$

(23) $$S=\frac{1}{N}\sum_{i=1}^{N}{\left(\frac{a_{i}-mean}{\sigma }\right)}^{3}$$

(24) $$K=\frac{1}{N}\sum_{i=1}^{N}{\left(a_{i}-mean\right)}^{4}/\sigma^{4}-3$$

(25) $$C_{\mathrm{xy}}=\mathrm{cov}(x,y)/\left(\sigma_{x}\sigma_{y}\right)$$

(26) $$\mathrm{SMA}=\sum_{i=1}^{N}\left(\left|x(i)\right|+\left|y(i)\right|+\left|z(i)\right|\right)$$

where *a_i_* is the acceleration data *I* = 1, 2, …, *N*. *N* is the number of samples, cov(*x*, *y*) is the covariance of the *x-* and *y*-axis acceleration. *x*(*i*), *y*(*i*) and *z*(*i*) respectively indicate the values of *x*-axis, *y*-axis and *z*-axis acceleration signals at the *i*th sampling point. After feature extraction, all features were normalized to the interval [0, 1]. Considering the balance of the data, the number of each activity sample of each dataset is as consistent as possible. The first column of Table 1 shows the activity performed by each subject and the right column shows the quantities of feature samples of different activities from the five body positions.

## 4. Experimental Setup

The experiments were implemented in Matlab 2014a using a computer with a 2.8 GHz processor and 6 GB memory. The parameters of DGSO are set as follows: population size *g* = 20, maximum iteration number *iter*_*max* = 100, fluorescein volatilization factor *ρ* = 0.4, fluorescein update rate γ = 0.5, dynamic decision domain update rate *β* = 0.06, initial fluorescein value *l*_0_ = 2, threshold *n_t_* = 5, initial dynamic decision radius $r_{d}^{i}(0)$ = 7, perceived radius *r_s_* = 12, *p*_1_ = 0.15, *p*_2_ = 0.75. All of these parameters were defined empirically. The leave-one-out (LOO) strategy was utilized to evaluate the proposed method. The data from four subjects was utilized as training data and half of the data from another remaining subject was utilized for selecting the ensemble with best performance. In addition, the other half of the data was utilized for testing the proposed approach. The verification was repeated 5 times until the data from all subjects had been utilized for pre-pruning and testing.

### 4.1. Pre-Pruning Based on Pairwise Diversity Measures

**Figure 6:**
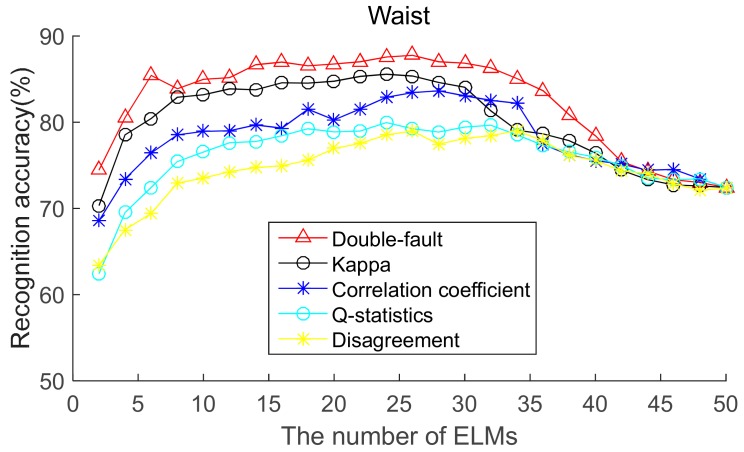
Recognition accuracy from waist position for ordered bagging according to five pairwise diversity measures.

**Figure 7:**
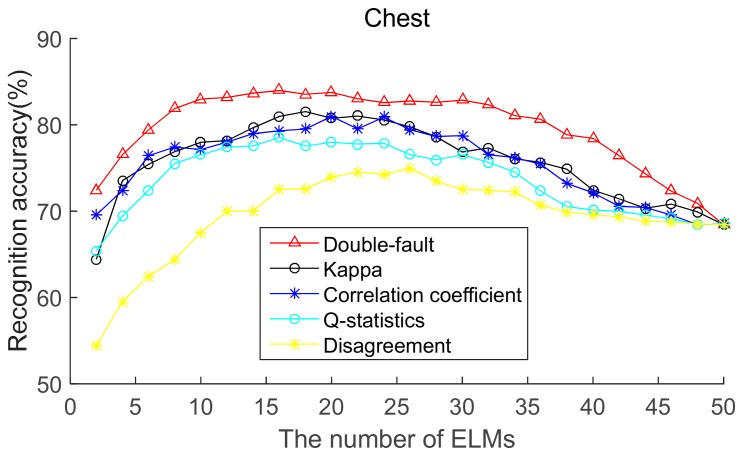
Recognition accuracy from chest position for ordered bagging according to five pairwise diversity measures.

**Figure 8:**
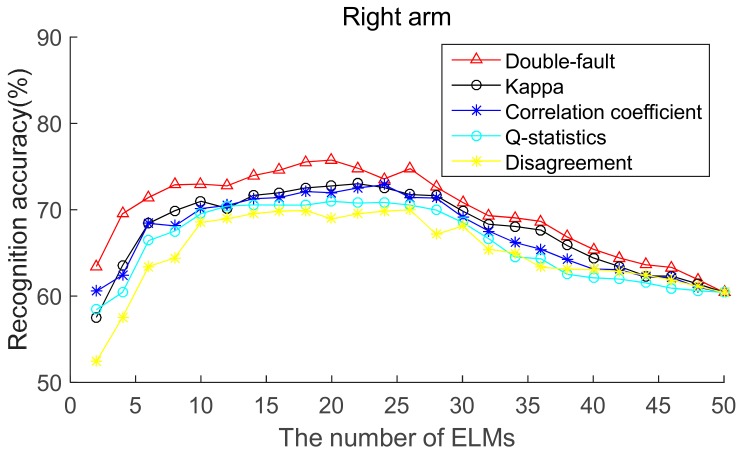
Recognition accuracy from right arm position for ordered bagging according to five pairwise diversity measures.

**Figure 9:**
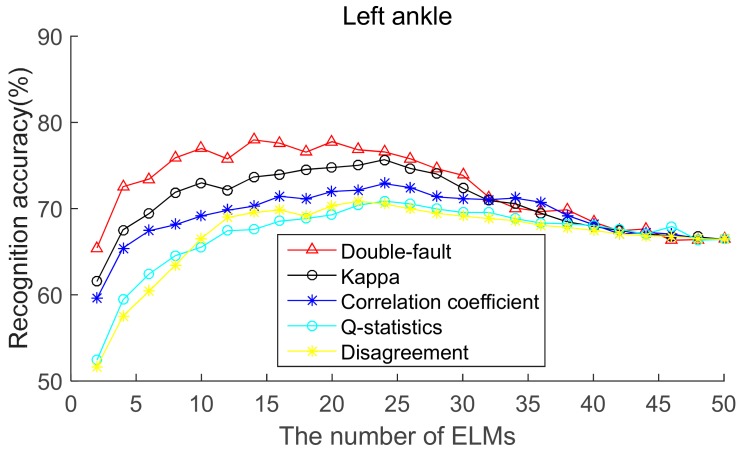
Recognition accuracy from left ankle position for ordered bagging according to five pairwise diversity measures.

**Figure 10:**
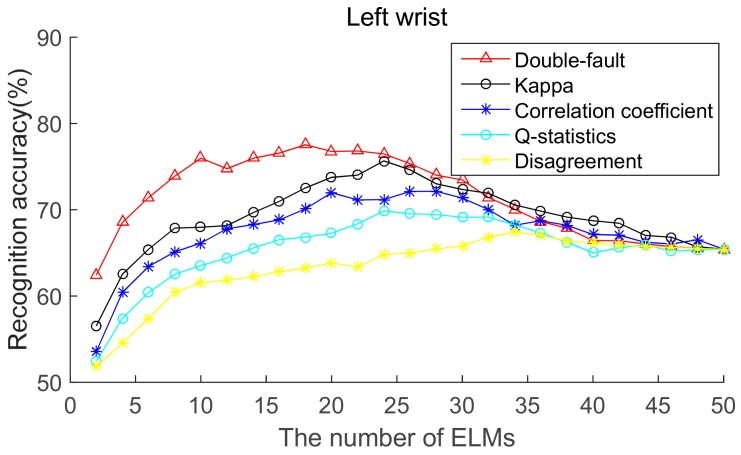
Recognition accuracy from left wrist position for ordered bagging according to five pairwise diversity measures.

To select the most effective method for pre-pruning the initial base classifier pool, according to diversity of base ELMs (from highest to lowest), the recognition accuracies of five body positions for ordered bagging based on initial pool with 50 base ELMs are shown in Figure 6, Figure 7, Figure 8, Figure 9 and Figure 10. It can be seen from Figure 6, Figure 7, Figure 8, Figure 9 and Figure 10 that, as there are few base classifiers in the initial stage of the ensemble, the ensemble system lacks diversity, which affects the ensemble’s accuracy. The ensemble accuracy reaches the maximum at an intermediate number of base ELMs. Then, there are a large number of redundant base classifiers in the ensemble system, which will result in a decrease in ensemble accuracy. Therefore, the ensemble accuracy increases first and then decreases with the number of base ELMs. This demonstrates that the ensemble accuracy can be improved by pre-pruning base ELMs with lower diversity with other base ELMs. Additionally, we can also find that the double-fault measure can achieve better results than the other four diversity measures with five sensor locations. Hence, the double-fault measure will be utilized for pre-pruning base ELMs in this paper.

The parameter *D*′ of the pre-prune is important, which determines the number of base classifiers in the GSO pruning. As we can see from Figure 6, Figure 7, Figure 8, Figure 9 and Figure 10, the number of base ELMs when the maximum ensemble accuracy obtained is different. It is unscientific to set fixed parameter *D*′ when data from different sensor positions and the number of base classifiers *D* are considered. Therefore, we set the parameter *D*′ according to statistical methods. Suppose *Div_i_* is the double-fault measure of the *i*th base ELM and [*Div*_1_, *Div*_2_, ……, *Div_D_*] represents the double-fault measure vector of *D* base classifiers, $\bar{Div}=\frac{1}{D}\sum_{i=1}^{D}Divi$ is the arithmetic mean of the double-fault measure of *D* base classifiers, we eliminate the base classifiers whose *Div_i_* is smaller than $\bar{D_{\mathrm{iv}}}$, and the remaining *D*′ base ELMs are utilized for the GSO-based selective ensemble. For initial pool with 50 base ELMs, the *D*′ is 18, 25, 27, 30, 29 for waist, chest, right arm, left ankle and left wrist, respectively.

### 4.2. Performance Measures

The accuracy measure is used to evaluate the performance of the proposed method, which can be expressed as follows:

(27) $$\mathrm{Accuracy}=\frac{TP+TN}{TP+TN+FP+FN}$$

where the variables *TP*, *TN*, *FP*, and *FN*, respectively, represent the number of true positive, true negative, false positive, and false negative outcomes in a given experiment.

In addition, F1 evaluation criteria are also considered. F1 is defined as the combination of precision and the recall, which are defined as follows:

(28) $$\mathrm{precision}=\frac{TP}{TP+FP}$$

(29) $$\mathrm{recall}=\frac{TP}{TP+FN}$$

The F1 is calculated as follows:

(30) $$F1=\frac{2\times \mathrm{recall}\times \mathrm{precision}}{\mathrm{recall}+\mathrm{precision}}$$

## 5. Experimental Results

To verify the proposed HAR approach, initial base classifier pools of different sizes (50, 100, 150, 200) were set up and utilized for the experiment. Table 2, Table 3, Table 4 and Table 5 show the comparative recognition performance of DMGSOSEN-based HAR approach with the best, average, and worst performance of the base ELMs in the initial pool. It can be observed from Table 2, Table 3, Table 4 and Table 5 that the performance obtained by the proposed approach is much better than the best and average performance of base ELMs in the initial base classifier pool. In addition, it can be found that no matter which position is considered, the proposed DMGSOSEN-based HAR approach does not achieve the best results when the size of initial base classifier pool is the largest. When the number of the initial base classifier is 100 and 150, the proposed approach is more likely to achieve better results. Furthermore, we also find that the recognition performances of the five positions are quite different, and the waist is more likely than the other four positions to achieve optimal recognition result.

Additionally, in order to gain a better insight into the activity recognition problem and the proposed DMGSOSEN base HAR method, the corresponding confusion matrix was constructed. Table 6, Table 7, Table 8, Table 9 and Table 10 show the results of using an initial pool of 100 base classifiers with the data from the five positions, respectively. We can observe that confusion occurs in most cases between activities such as (GU, GD), (W, R), (J, GD) and (J, GU), especially when the data from the right arm, left ankle and left wrist were utilized. Furthermore, we find that data from the wrist and chest are superior to other positions for recognizing similar activities, such as (GU, GD) and (W, R). In addition, we can also observe that no matter which position is utilized, the activity standing (S) is much easier to recognize than activities such as running (R), going upstairs (GU) and going downstairs (GD).

### 5.1. Compared to Traditional Ensemble Algorithm-Based HAR

In addition, several comparative experiments were carried out to evaluate the proposed approach in comparison with the traditional ensemble methods Bagging and Adaboost. Table 11, Table 12, Table 13 and Table 14 show the comparative results with initial pools of different sizes. In Table 11, Table 12, Table 13 and Table 14, *n* represents the number of base classifiers selected by the proposed method. It can be seen from Table 11, Table 12, Table 13 and Table 14 that although the ensembles derived using the traditional methods Bagging and Adaboost have more base classifiers, the proposed DMGSOSEN outperforms these two methods with fewer base classifiers, which shows that it may be better to derive ensembles with many base classifiers than with all. Furthermore, we find that the proposed method eliminates more than 60% of the base classifiers in the initial pool and achieves better recognition performance compared with Bagging and Adaboost, thus demonstrating the effectiveness of the proposed DMGSOSEN for HAR.

### 5.2. Compared to the State-of-the-Art Pruning Approach-Based HAR

To better assess the performance of the proposed DMGSOSEN-based HAR approach, we utilized an initial pool containing 100 base ELMs in order to compare it with other state-of-the-art pruning method-based HAR. These pruning methods included aggregation ordering in bagging (AGOB) [56], ordered bagging ensemble (POBE) [57], D-D-ELM [58], DF-D-ELM [59], GASEN [31], MOAG [60], RRE [61], and DivP [38]. Among these, AGOB, POBE and MOAG are all studies of ordering-based selective ensembles, and the basic classifiers are ordered by using their proposed metrics. GASEN utilizes GA to optimize the weights of the base classifier and the combination of base classifiers with the best performance constitutes the final ensemble. RRE attempts to make full use of the votes of the worst single model in the ensemble. DivP applies GA to combine five pairwise diversity matrices, utilizing a graph coloring method to generate candidate ensembles. DD-ELM and DF-D-ELM attempts to remove the base ELMs by using the disagreement measure and the double-fault measure, respectively.

Table 15, Table 16 and Table 17 show the comparative results of these methods at the five sensor locations. It can be seen from the Table 15, Table 16 and Table 17 that the proposed selective ensemble HAR method based on DMGSOSEN achieves the best recognition performance when compared with the other algorithms. Although the number of base classifiers selected by the proposed method is slightly more than the DivP, it can achieve better performance than DivP with data of five sensor locations. Overall, the recognition performance and the number of base classifiers demonstrate that the proposed DMGSOSEN-based HAR approach performs better than other selective ensemble approaches, which indicates that the DMGSOSEN method has stronger generalization ability and learning efficiency in HAR tasks.

### 5.3. Compared to the Previous Studies in HAR

To further evaluate the performance of this study, we compared it with some previous studies in HAR, including EEMD+FS+SVM [12], ACELM [17], CELearning [41], tFFT+Convnet [62] and KPCA+DBN [63]. These studies, conducted in recent years, include deep learning, ensemble learning and feature selection for HAR. The methods and results of all these studies are shown in Table 18. Although these studies are based on their different datasets and methods, we can know the relative performance of this research in the field of HAR. It is obvious that our study has the best performance compared with other previous studies. We can achieve 96.7% recognition accuracy and F1 score by using our proposed base ensemble ELM approach.

## 6. Conclusions

Traditional HAR systems based on a single classifier are likely to perform poorly due to the diversity of activity styles. Combining multiple classifiers appears to be a very effective approach for improving the performance and generalization ability of the HAR system. However, there would be some base classifiers that are redundant and perform poorly in multiple classifier systems, providing no contribution to the performance of the HAR system. To tackle this issue, a HAR approach based on ELM and DMGSOSEN is proposed in this paper. The DMGSOSEN is a novel ensemble pruning method using a combination of existing algorithms for ensemble learning-based HAR. Compared to the other four pairwise diversity measures, the double-fault measure shows better performance for pre-pruning the initial pool on five sensor locations. The experimental results on the dataset with five positions show that the DMGSOSEN-based HAR approach can achieve better recognition performance with fewer base ELMs compared with traditional ensemble HAR methods: Bagging, Adaboost and other state-of-the-art pruning-based HAR methods.

In future work, more complex activities will be added to test the proposed method, and we will optimize the module’s performance by considering other state-of-art machine learning methods, such as deep leaning. For example, when determining base classifiers, kernel extreme learning machine (KELM) is an improvement of ELM with characteristics of fast training and good generalization. In addition, more combinations of diversity measures and heuristic searching algorithms such as particle swarm optimization or fish swarm algorithm will be attempted to search for a sub-ensemble for constructing a selective ensemble-based HAR system.

The dataset utilized in this work only contains six daily activities from five subjects, who were all healthy with similar ages. This is a limitation of this work. In future works, we will attempt to collect data from more subjects with different living behaviors, ages, genders, etc., and more high-level activities (open door, cooking, etc.) will be considered in order to verify the proposed method. Furthermore, some public datasets should be utilized to test the performance of the proposed method and compare it with some state-of-the-art approaches. Moreover, this study is also limited due to the lack of a validation set completely different from the training set. We will utilize datasets with different ages or physical characteristics to test the applicability of the proposed method.

## Figures and Tables

**Table 1:** Same acquisition feature samples of different activities from the five body positions.

Activity	Feature Samples
Walking (W)	6164
Running (R)	6028
Going upstairs (GU)	5772
Going downstairs (GD)	5836
Jumping (J)	5982
Standing (S)	6043

**Table 2:** Performance of the ensembles for initial pool sizes of 50 (Accuracy/F1%).

Position	50			
	DMGSOSEN	Best	Mean	Worst
waist	95.9/95.8	85.5/85.9	74.6/74.6	60.5/60.3
chest	94.4/94.4	82.4/82.6	72.1/72.3	52.7/52.9
right arm	91.8/91.7	77.3/77.1	69.6/69.8	50.5/50.2
left ankle	90.2/90.4	79.6/79.2	70.2/70.2	49.6/49.9
left wrist	89.1/89.2	78.7/78.5	70.8/70.4	49.7/49.4

**Table 3:** Performance of the ensembles for initial pool sizes of 100 (Accuracy/F1%).

Position	100			
	DMGSOSEN	Best	Mean	Worst
waist	96.7/96.7	88.9/88.8	74.5/74.4	60.2/60.5
chest	95.7/95.7	84.8/84.8	72.4/72.3	51.8/51.8
right arm	92.4/92.4	81.5/81.2	70.1/70.4	49.7/47.9
left ankle	90.5/90.4	83.7/83.4	70.6/70.3	49.3/49.5
left wrist	89.3/89.3	84.1/84.5	71.2/71.5	49.1/49.3

**Table 4:** Performance of the ensembles for initial pool sizes of 150 (Accuracy/F1%).

Position	150			
	DMGSOSEN	Best	Mean	Worst
waist	95.5/95.4	89.1/89.4	74.1/74.4	60.3/60.4
chest	95.2/95.1	84.9/84.6	72.4/72.6	50.7/50.7
right arm	92.1/92.2	82.6/82.6	69.7/69.8	49.4/49.5
left ankle	90.3/90.4	84.2/84.1	71.4/71.9	48.5/48.7
left wrist	89.2/89.3	84.9/84.9	70.9/70.7	47.2/47.2

**Table 5:** Performance of the ensembles for initial pool sizes of 200 (Accuracy/F1%).

Position	200			
	DMGSOSEN	Best	Mean	Worst
waist	94.2/94.3	89.8/89.8	74.6/74.5	60.1/60.3
chest	93.9/93.5	85.9/85.9	72.5/72.7	49.2/49.4
right arm	92.7/92.5	83.8/83.9	69.6/69.4	47.7/47.6
left ankle	90.1/90.2	85.2/85.2	71.6/71.5	46.6/46.7
left wrist	88.9/88.9	84.3/84.4	70.3/70.3	45.7/45.7

**Table 6:** Confusion matrix for DMGSOSEN-based HAR on the data from the waist when the initial pool size is 100.

	W	R	GU	GD	J	S
W	600	9	5	4	2	2
R	7	576	8	6	4	2
GU	4	8	550	7	8	1
GD	2	6	11	562	7	1
J	0	2	2	3	575	0
S	3	1	1	1	2	598

**Table 7:** Confusion matrix for DMGSOSEN-based HAR on the data from the chest when the initial pool size is 100.

	W	R	GU	GD	J	S
W	592	11	9	3	3	2
R	12	572	12	8	7	2
GU	6	6	541	11	8	3
GD	4	8	12	556	5	4
J	1	3	2	4	573	1
S	1	2	1	1	2	592

**Table 8:** Confusion matrix for DMGSOSEN-based HAR on the data from the right arm when the initial pool size is 100.

	W	R	GU	GD	J	S
W	577	17	16	14	8	10
R	14	549	10	18	12	3
GU	11	16	532	12	13	2
GD	6	11	10	523	11	6
J	4	4	6	12	547	3
S	4	5	3	4	7	580

**Table 9:** Confusion matrix for DMGSOSEN-based HAR on the data from the left ankle when the initial pool size is 100.

	W	R	GU	GD	J	S
W	567	21	16	21	16	6
R	14	535	19	17	9	2
GU	15	18	517	23	13	4
GD	10	18	15	503	19	3
J	6	7	14	15	538	9
S	4	3	2	4	3	580

**Table 10:** Confusion matrix for DMGSOSEN-based HAR on the data from the left wrist when the initial pool size is 100.

	W	R	GU	GD	J	S
W	562	36	12	17	7	7
R	13	528	21	21	17	2
GU	10	9	514	24	22	12
GD	26	20	22	495	16	7
J	2	5	6	22	533	11
S	3	4	2	4	3	565

**Table 11:** Performance comparison with Adaboost and Bagging on 50 ELMs (Accuracy/F1%).

Position	50					
	Adaboost	*n*	Bagging	*n*	DMGSOSEN	*n*
waist	88.2/88.4	50	86.4/86.5	50	95.9/95.8	9
chest	85.8/85.6	50	84.1/84.2	50	94.4/94.4	11
right arm	79.5/79.4	50	78.4/78.4	50	91.8./91.7	14
left ankle	83.8/83.7	50	82.4/82.3	50	90.2/90.4	17
left wrist	84.8/84.7	50	83.5/83.4	50	89.1/89.2	15

**Table 12:** Performance comparison with Adaboost and Bagging on 100 ELMs (Accuracy/F1%).

Position	100					
	Adaboost	*n*	Bagging	*n*	DMGSOSEN	*n*
waist	88.6/88.5	100	86.9/86.9	100	96.7/96.7	16
chest	86.4/86.2	100	85.2/85.3	100	95.7/95.7	19
right arm	79.7/79.8	100	79.6/79.6	100	92.4/92.4	25
left ankle	84.2/84.3	100	83.3/83.4	100	90.5/90.4	22
left wrist	84.9/84.9	100	84.2/84.3	100	89.3/89.3	23

**Table 13:** Performance comparison with Adaboost and Bagging on 150 ELMs (Accuracy/F1%).

Position	150					
	Adaboost	*n*	Bagging	*n*	DMGSOSEN	*n*
waist	89.1/89.2	150	87.3/87.2	150	95.5/95.4	31
chest	86.2/86.2	150	85.4/85.3	150	95.2/95.1	33
right arm	79.8/79.9	150	78.8/78.9	150	92.1/92.2	42
left ankle	84.8/84.6	150	83.5/83.3	150	90.3/90.4	38
left wrist	84.9/84.8	150	84.4/84.2	150	89.2/89.3	36

**Table 14:** Performance comparison with Adaboost and Bagging on 200 ELMs (Accuracy/F1%).

Position	200					
	Adaboost	*n*	Bagging	*n*	DMGSOSEN	*n*
waist	89.4/89.5	200	87.2/87.3	200	94.2/94.3	38
chest	86.8/86.7	200	85.1/85.2	200	93.9/93.5	43
right arm	79.6/79.4	200	79.4/79.4	200	92.7/92.5	52
left ankle	84.3/84.4	200	83.8/83.7	200	90.1/90.2	48
left wrist	84.2/84.2	200	83.5/83.6	200	88.9/88.9	51

**Table 15:** Performance comparison (Accuracy/F1%) and number of ELMs after pruning achieved by comparative algorithms.

Position	DMGSOSEN	*n*	AGOB	*n*	POBE	*n*
waist	96.7/96.7	16	88.6/88.6	26	85.2/85.3	29
chest	95.7/95.7	19	86.5/86.4	32	84.6/84.6	35
right arm	92.4/92.4	25	80.3/80.3	38	79.4/79.4	38
left ankle	90.5/90.4	22	84.6/84.5	34	84.2/84.2	35
left wrist	89.3/89.3	23	82.8/82.3	35	81.7/81.7	32

**Table 16:** Performance comparison (Accuracy/F1%) and number of ELMs after pruning achieved by comparative algorithms.

Position	D-D-ELM	*n*	DF-D-ELM	*n*	GASEN	*n*
waist	89.3/89.5	50	89.7/89.6	48	87.4/87.5	36
chest	88.5/88.5	50	85.3/85.3	52	84.5/84.5	43
right arm	81.3/81.4	50	74.5/75.4	57	76.6/76.6	38
left ankle	84.3/84.3	50	82.5/82.5	51	85.2/85.3	41
left wrist	82.9/82.9	50	81.8/81.8	51	84.7/84.7	44

**Table 17:** Performance comparison (Accuracy/F1%) and number of ELMs after pruning achieved by comparative algorithms.

Position	MOAG	*n*	RRE	*n*	DivP	*n*
waist	81.2/81.3	27	83.3/83.4	19	89.4/89.3	11
chest	80.6/80.6	29	82.4/82.4	23	87.3/87.3	17
right arm	75.3/75.2	35	74.5/74.5	31	80.2/80.2	23
left ankle	78.4/78.4	25	79.8/79.8	24	84.3/84.1	22
left wrist	76.2/76.3	28	79.1/79.2	26	83.2/83.3	20

**Table 18:** Comparison with some previous studies in HAR.

Author	Method	Performance (ACC/F1%)
Wang et al. [12]	EEMD+FS+SVM	81.2/-
Yuan et al. [17]	ACELM	95.02/-
Xu et al. [41]	CELearning	95.1/-
Ronao et al. [62]	tFFT+Convnet	95.75/-
Hassan et al. [63]	KPCA+DBN	95.85/-
Our proposed method	ELM+DMGSOSEN	96.7/96.7
